# Cardioprotective Effects of Sphingosine-1-Phosphate Receptor Immunomodulator FTY720 in a Clinically Relevant Model of Cardioplegic Arrest and Cardiopulmonary Bypass

**DOI:** 10.3389/fphar.2019.00802

**Published:** 2019-07-18

**Authors:** Naseer Ahmed, Adeela Mehmood, Daniele Linardi, Soban Sadiq, Maddalena Tessari, Sultan Ayoub Meo, Rehana Rehman, Waseem M. Hajjar, Nazeer Muhammad, Muhammad Perwaiz Iqbal, Anwar-ul-Hassan Gilani, Giuseppe Faggian, Alessio Rungatscher

**Affiliations:** ^1^Department of Biological and Biomedical Sciences, The Aga Khan University, Karachi, Pakistan; ^2^Department of Surgery, Cardiac Surgery Division, University of Verona Medical School, Verona, Italy; ^3^Department of Pharmacology, Liaqat National Medical College, Karachi, Pakistan; ^4^Pharmacology and Molecular Lab, University of Liverpool, United Kingdom; ^5^Department of Physiology, College of Medicine, King Saud University, Riyadh, Saudi Arabia; ^6^Department of Thoracic Surgery, College of Medicine, King Saud University, Riyadh, Saudi Arabia; ^7^Department of Mathematics, COMSATS University Islamabad, Wah Campus, Pakistan

**Keywords:** cardioplegic arrest, cardioprotection, ischemia reperfusion injury, anti-inflammatory, FTY 720

## Abstract

**Objective:** FTY720, an immunomodulator derived from sphingosine-1-phosphate, has recently demonstrated its immunomodulatory, anti-inflammatory, anti-oxidant, anti-apoptotic and anti-inflammatory properties. Furthermore, FTY720 might be a key pharmacological target for preconditioning. In this preclinical model, we have investigated the effects of FTY720 on myocardium during reperfusion in an experimental model of cardioplegic arrest (CPA) and cardiopulmonary bypass.

**Methods:** 30 Sprague–Dawley rats (300–350 g) were randomized into two groups: Group-A, treated with FTY720 1 mg/kg *via* intravenous cannulation, and Group-B, as control. After 15 min of treatment, rats underwent CPA for 30 min followed by initiation of extracorporeal life support for 2 h. Support weaning was done, and blood and myocardial tissues were collected for analysis. Hemodynamic parameters, inflammatory mediators, nitro-oxidative stress, neutrophil infiltration, immunoblotting analysis, and immunohistochemical staining were analyzed and compared between groups.

**Results:** FTY720 treatment activated the Akt/Erk1/2 signaling pathways, reduced the level of inflammatory mediators, activated antiapoptotic proteins, and inhibited proapoptotic proteins, leading to reduced nitro-oxidative stress and cardiomyocyte apoptosis. Moreover, significant preservation of high-energy phosphates were observed in the FTY720-treated group. This resulted in improved recovery of left ventricular systolic and diastolic functions.

**Conclusion:** The cardioprotective mechanism in CPA is associated with activation of prosurvival cell signaling pathways that prevents myocardial damage. FTY720 preserves high-energy phosphates attenuates myocardial inflammation and oxidative stress, and improves cardiac function.

**Figure:**
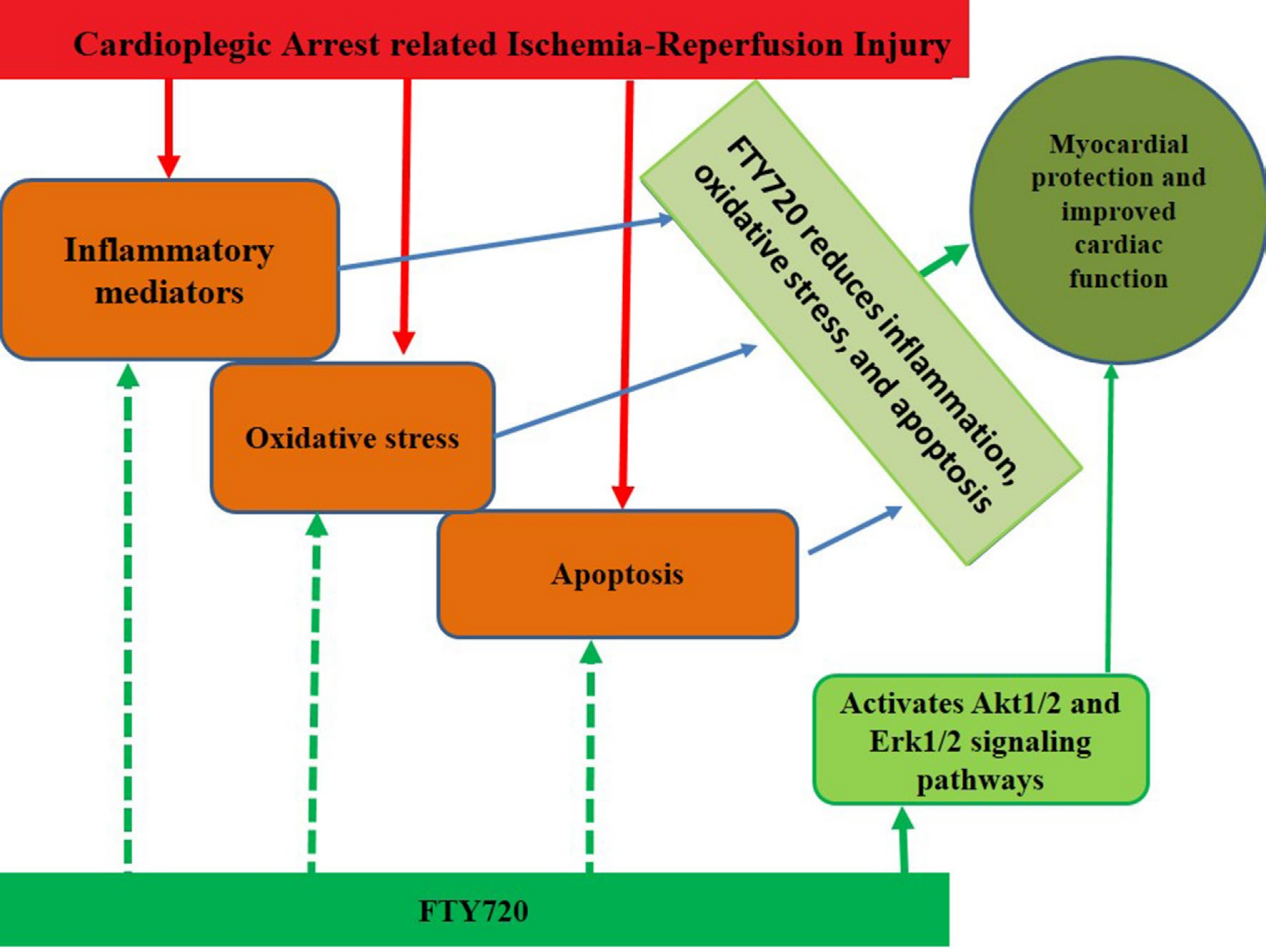
Graphical Abstract

## Introduction

Fingolimod (FTY720), an immunomodulator drug targeting the sphingosine-1-phosphate (S1P) receptor with anti-inflammatory properties, is a widely recommended drug for relapsing-remitting multiple sclerosis (Pelletier and Hafler, 2012; Ayzenberg et al., 2016). It has been demonstrated to protect the heart against myocardial injury in various animal models (Kennedy et al., 2009; Santos-Gallego et al., 2016; Ahmed, 2017; Ahmed et al., 2017). Mainly, these protective effects were attributed to its anti-oxidant, anti-apoptotic and anti-inflammatory properties (Santos-Gallego et al., 2016; Ahmed et al., 2017). Oxidative stress and inflammatory mediators are known to be associated with ischemia–reperfusion (I/R) injury and most probably are responsible for myocardial injury (Penna et al., 2009; Sanada et al., 2011)

Furthermore, both inflammatory mediators and oxidative stress trigger the opening of the mitochondrial permeability transition pore (mPTP), which happens in reperfusion injury (Venditti et al., 2001). The mPTP opening causes myocardial damage by initiating the uncoupling of mitochondria, leading to hydrolysis instead of ATP synthesis.

Therapeutic targets that play a role in the inhibition of mPTP opening are considered cardioprotective, and as per literature, cardiac functional recovery can be estimated by a reduction in mPTP opening (Griffiths and Halestrap, 1995; Javadov et al., 2017). Recent studies have demonstrated that along with other inflammatory cascades and oxidative stress, nitroxidative stress also plays an important role in I/R-induced myocardial tissue injury (Plotnikov et al., 2007). Excessive production of nitric oxide (NO) from NOS and its highly reactive products such as peroxynitrite and superoxide has been reported to activate several apoptotic pathways leading to apoptotic cell death (Gielis et al., 2017). Furthermore, FTY720 also play important role in activating multiple signaling pathways including Akt/Erk1/2 and Pak1 to reduce IRI, cardiac arrhythmias and ventricular hypertrophy (Wang et al., 2018; Wang et al., 2019).

Although there is evidence that FTY720 contributes to cardioprotection against I/R injury, its protective role in association with cardioplegia during cardiac surgery has not been studied yet.

The S1P receptor agonist (FTY720) might act as a potential pharmacological agent for cardioprotection in cardioplegia; therefore, the aim of this study was to assess the cardioprotective effects of S1P receptor activation by FTY720 in the clinical setting of cardioplegic arrest (CPA) in an experimental rat model. To understand the cardioprotective role of FTY720, we investigated inflammatory mediators and oxidative stress in addition to functional hemodynamic parameters.

## Methods

This study was conducted in the Division of Cardiac Surgery and Translational Surgery Lab, University of Verona Medical School, Verona, Italy. The study was directed in compliance with best research practice and according to the Declaration of Helsinki principles. The protocol was approved by the local Joint Ethical Committee for University of Verona and Hospitals (Verona and Rovigo), Italy (BBCCH1337).

### Animals

After institutional animal care committee approval, 30 male Sprague–Dawley rats (300–350 g) were obtained from Harlan Laboratories (Udine, Italy) and fed on standard rat chow, which they had access *ad libitum*. The rats were housed at a density of 3–4 per cage and maintained on a 12-h light/dark cycle at 21°C.

Rats were secured in a supine position on a heating board to maintain rectal temperature at 37°C during the surgical procedure before the initiation of CPA. Rats were anesthetized (sodium pentobarbital, 30 mg/kg intraperitoneally) and intubated using oropharynx with a 14-gauge polyethylene tube. Mechanical ventilation was done with a rodent ventilator (Harvard Apparatus Inc., Holliston, MA). The tidal volume was 6 ml/kg, and the respiratory rate was 50–60 breaths/min with an air–oxygen mixture (inspired oxygen fraction = 0.5) (Rungatscher et al., 2015). The left femoral artery was cannulated with a heparinized 24-gauge Teflon catheter to monitor the systemic arterial pressure, and central cannulation was performed, as previously described (Rungatscher et al., 2013). Briefly, after complete sternotomy, a venous cannula (a modified 4-hole 16-gauge Angiocath catheter) was introduced into the right atrium with proper drainage. The left common carotid artery was cannulated using an 18-gauge catheter, advancing to the aortic arch.

Full heparinization (500 IU/kg) was ensured after surgical preparation and immediately before cardiopulmonary bypass and CPA initiation to reduce overall blood loss. The setup consisted of a venous reservoir, a roller pump, a hollow fiber oxygenator (Sorin, Mirandola, Italy), and a vacuum regulator with an applied pressure of −30 mm H_2_O to facilitate venous drainage, all connected by a 1.6-mm internal diameter plastic tubing. Total priming volume was 10.5 ml, and gas exchange surface and heat exchange surface were of 450 cm^2^ and 15.8 cm^2^, respectively.

### Experimental Design

**Figure 1:**
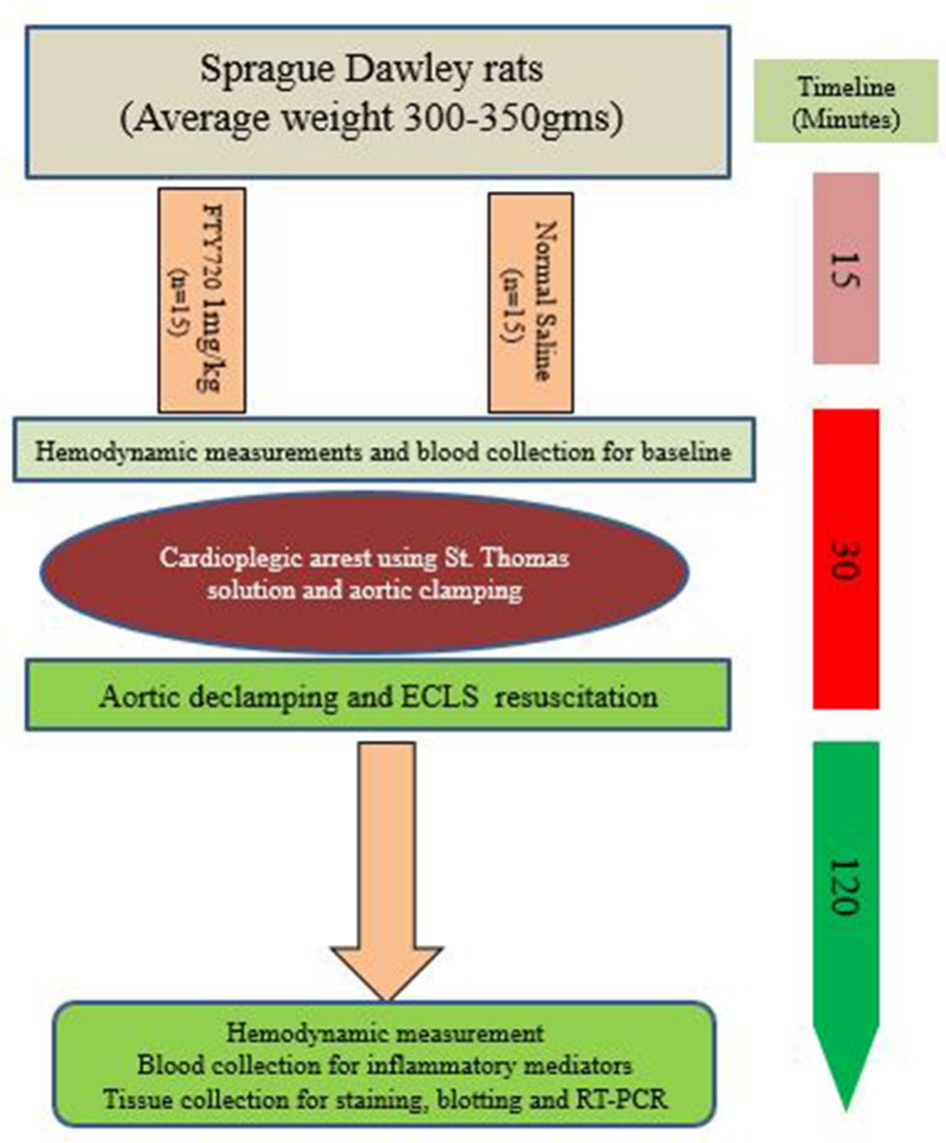
Schematic view of experimental design.

In this study, the rats were randomized into two groups (A and B): Group A was treated with FTY720 at a dose of 1 mg/kg intravenously and Group B with normal saline. After 15 min of both treatments, rats from both groups underwent CPA using cold St. Thomas solution and aortic clamps for 30 min after initiation of extra corporeal life support (ECLS) for the next 2 h, as described in a previous study (Rungatscher et al., 2013). During ECLS, internal temperature was kept at 36–37°C in both groups. After 2 h of reperfusion, ECLS weaning was done in all rats, and blood and tissue were harvested for further analysis. A schematic review of the experiment is illustrated in **Figure 1**.

### Hemodynamics

Hemodynamic parameters were measured at baseline and after ECLS weaning using a 2-Fr microtip pressure–volume (P-V) conductance catheter (SPR-838, Millar Instruments, Houston, TX) inserted into the right carotid artery and advanced into the left ventricle. Signals were recorded at a sampling rate of 1,000 samples using a P-V conductance system (MPVS-400, Millar Instruments), stored and displayed on a personal computer by the PowerLab Chart 5 Software System (AD Instruments, Colorado Springs, CO). Hemodynamics were measured by recording the left ventricular end-systolic (LVESP) and end-diastolic (LVEDP) pressures, along with contractility index measuring dP/dt min and dP/dt max (Rungatscher et al., 2015).

### Serum Inflammatory Markers Analysis

The levels of inflammatory mediators, tumour necrosis factor (TNF)-α, interleukin (IL)-6, intercellular adhesion molecule (ICAM)-1, and IL-1β (Thermo Scientific, Rockford, IL), were determined by enzyme-linked immunosorbent assay (ELISA) kits according to the manufacturer’s instructions (Ahmed et al., 2017).

### Evaluation of Nitro-Oxidative Stress

To investigate malondialdehyde (MDA) levels in myocardial tissue, samples were processed as described by Ohkawa et al. (1979). After centrifugation, supernatant (0.5 ml) reacts with thiobarbituric acid (TBA) (0.67%) (1 ml) using another 0.5 ml of trichloroacetic acid (TCA) (20%). The myocardial tissue samples were incubated at 37°C for 15 min in a water bath and then allowed to cool. The color compound obtained from the supernatant was extracted using n-butanol, and separation was done by centrifugation at 3,000 rpm for 15 min. Absorbance was measured at 532 nm (Jenway 6305 UV/visible spectrophotometer). The MDA levels in the hearts were expressed in nmol/g of myocardial tissue (Ohkawa et al., 1979).

The analysis of reactive oxygen species (ROS) was performed with a photometric analytical system as explained by Rizzo et al. (2008). This procedure measures the ROS that reacts with a particular chromogen, leading to the formation of a colored compound in the presence of a buffered solution. This compound can be measured photometrically (absorbance peak at 505 nm maximum). The determined absorbance value is expressed as Carr. units (1 U. Carr = 0.08 mg/100 ml H_2_O_2_) (Rizzo et al., 2008).

To assess nitrogen ROS, peroxynitrites were analyzed by measuring the nitrosylation of cardiac proteins using an antibody against nitrotyrosine by immunohistochemistry using a protocol described previously (Ahmed et al., 2017).

### Immunoblotting Analysis

The chemical reagents and SDS-PAGE apparatus were obtained from Bio-Rad (Hercules, CA). The apparatus for immunoblotting was purchased from SciePlas (Southam, England). The rabbit antibodies for phospho-specific Akt1/2 and ERK1/2 goat anti-rabbit IgG conjugated with horseradish peroxidase (HRP) were purchased from Abcam (Cambridge, MA). The rabbit anti-phosphorylated Akt1/2 and ERK1/2 antibodies were purchased from Cell Signaling (Beverly, MA). The remaining reagents were purchased from Sigma Chemical (St. Louis, MO).

The myocardial tissue was homogenized in buffer solution containing 1% Triton with phosphatase and protease inhibitors cocktail from Sigma Chemical, as described by Giani et al. (2007). Tissue extracts were centrifuged at 16,000 rpm for 15 min at 4°C, and supernatants were collected in separate aliquots. Protein concentration in the extract was determined by using the BCA assay kits (Beyotime Institute of Biotechnology, China) according to the manufacturer’s instructions and vector analysis. To assess phosphorylation levels of Akt1/2 and ERK1/2, equal amounts of solubilized proteins (35 μg) were denatured by boiling for 5 min at 100°C in reducing buffer, resolved by SDS-PAGE. The resolved proteins were transferred to polyvinylidenedifluoride (PVDF) membranes, pre-wetted with methanol and distilled water, before submersion in 30% methanol containing transfer buffer. After transfer, membranes were incubated for 1 h in blocking solution (5% fetal bovine serum in TBS containing 0.1% Tween 20), followed by incubation with primary antibodies (1:1,000 in blocking solution) overnight at 4°C with both unphosphorylated and phospho-specific antibodies. Equal loading of protein in the acrylamide gels was confirmed by reusing the PVDF membranes with antibody against an anti-glyceraldehyde-3-phosphate dehydrogenase (anti-GAPDH). After overnight incubation in primary antibody, membranes were washed generously with TBS-Tween 20 solution for 20 min. Washed membranes were incubated with secondary HRP-conjugated antibodies at a dilution of 1:10,000, and HRP-substrate chemiluminescence was used through Syngene Western blotting detection system. The quantification of protein band densities was analyzed by ImageJ 1.37 software (Giani et al., 2007).

### Immunohistochemical Staining

Myocardial tissue sections (3 microns thick) on polarized slides were heated to 60°C for 1 h and rehydrated for 20 min with xylene and graded ethanol solution. Antigen retrieval was done with 0.01 M citrate buffer at pH 6.0 for 30 min in a water bath. Gradual cooling was allowed for the next 15 min, followed by sequential rinsing with tap water and phosphate buffered solution (PBS). Endogenous peroxidase activity was suppressed by TBS-T solution with 3% hydrogen peroxide incubation. All sections were incubated overnight at 4°C in primary antibodies, Bax (Abcam, Cambridge, United Kingdom), Bcl-2 (Dako, Glostrup, Denmark), and nitrotyrosine (Sigma Aldrich, United Kingdom). After thorough washing of primary antibodies, the slides were incubated with biotin-conjugated secondary antibody for 30 min and di-amino benzidine and hydrogen peroxide chromogen substrate (Dako Corp). All slides were counterstained with hematoxylin and mounted. The negative controls were incubated with non-immune rabbit IgG. Tissue sections from each animal were imaged using a light microscope Nikon E400 (Nikon Instrument Group, Melville, NY) (Ahmed et al., 2017).

### Bcl-2 and Bax Expression Measurement Using Real Time-Polymerase Chain Reaction

The RNA extraction was done from myocardial tissue and was assessed for quality and concentration. cDNA was generated from RNA by reverse transcription kit, according to manufacturer’s guidelines. The Bcl-2, Bax, and GAPDH (housekeeping gene) primers (**Table 1**) were used to determine the mRNA expression by real-time fluorescence-based quantitative PCR. Conditions followed for this were at 94°C for 5 min, then 45 cycles at 94°C for 30 s, 57°C for 30 s, 75°C for 30 s, and finally 72°C for 5 min Data­ were exported to MS Excel sheet for further analysis. Relative quantification was calculated using the 2^–ΔC^ ^t^ model, where ΔCt (Target gene) = CT (Target gene) – CT (Ref. gene) (Li et al., 2018).

**Table 1:** RT-PCR primer sequences.

Gene name	Primer sequences
Bax	F 5′-AGACACCTGAGCTGACCTTGGAG-3′
	R 5′-GTTGAAGTTGCCATCAGCAAACA-3′
Bcl-2	F 5′-TGAACCGGCATCTGCACAC-3′
	R 5′-CGTCTTCAGAGACAGCCAGGAG-3′
GAPDH	F 5′-ATGGCACCGTCAAGGCTGAG-3′
	R 5′-TGTCAGGTACGGTAGTGACG-3′

RT-PCR, Real Time-Polymerase Chain Reaction.

### High-Energy Phosphates Measurement

The high-energy phosphates estimation includes the determination of adenosine nucleotides, that is, adenosine monophosphate (AMP), adenosine diphosphate (ADP), adenosine triphosphate (ATP), and phosphocreatine (PCr). Myocardial tissue was clamped from the beating heart in the chest using liquid nitrogen precooled clamps. The frozen ball of myocardial tissue was stored at −80°C until extract preparation. Collected heart tissue was submerged in liquid nitrogen, and frozen tissue was weighed (50–100 mg) and deproteinized by homogenization using 500 µl of 0.4 mol/L perchloric acid in a ball mill (Braun, Melsungen, Germany). The 150 µl of the acid extract was centrifuged at 12,000 G, followed by neutralization with 2 mol/L potassium carbonate at 4°C. The potassium carbonate was centrifuged, and the supernatant was stored at −28°C till further use.

Chromatographic separation was performed on a Hypersil ODS column (5 μm, 250 mm long × 4 mm ID) with an AS-100 HRLC automatic sampling system (Bio-Rad), a 127 HPLC solvent module, and a diode array detector module (Beckman). Detector signals (absorbance at 214 nm for PCr and 254 nm for adenine dinucleotide) were recorded with an AGC personal computer. System Gold (Beckman) was used as controller for data requisition and analysis. The extract pellets were dissolved in 1 ml of 0.1 mol/L sodium hydroxide and further diluted 1:10 with physiological saline for protein determination (BCA Protein Assay, Pierce) (Hallstrom et al., 2002).

### Statistical Analysis

Data analysis was done using SPSS software version 21 (SPSS Inc., Chicago, Illinois) in all the treatment and control groups. Mean ± SD was calculated for all the measurements and appropriate statistical tests, that is, Student *t* test or Mann–Whitney nonparametric tests, were applied to compare the two groups. A *p* value less than 0.05 was considered statistically significant.

## Results

### Effect of FTY720 on Left Ventricular Function

**Figure 2:**
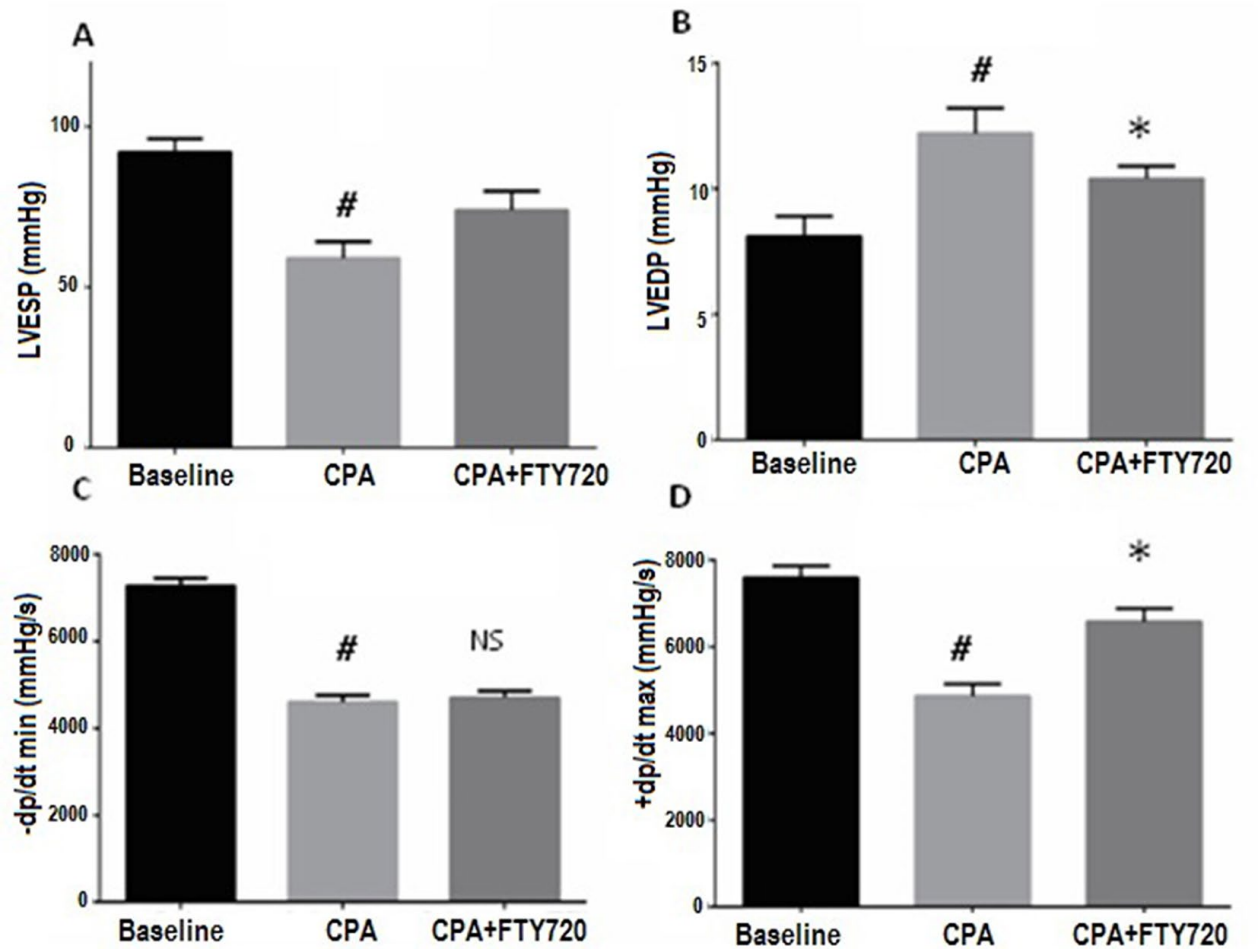
Hemodynamic parameters measured at baseline and 2 h after reperfusion. Effects of FTY720 on left ventricular function in the rats with CPA-reperfusion-induced injury. Treatment (FTY720 or saline) was done 15 min before cardioplegic arrest. **(A)** Effects of FTY720 on LVESP. **(B)** Effects of FTY720 on LVEDP. **(C)** Effects of FTY720 on LV dP/dt max. **(D)** Effects of FTY720 on LV dP/dt min. LVESP and LVEDP were measured using a multichannel physiological recorder. LV dP/dt max and dP/dt min were expressed as mmHg/s. LVESP and LVEDP were expressed as mmHg. LV dP/dt max, rate of maximum positive left ventricular pressure development; LV dP/dt max, rate of maximum negative left ventricular pressure development; LVESP, left ventricular end-systolic pressure; LVEDP, left ventricular end-diastolic pressure. Values are expressed as the means ± SD (*n* = 15 in each group). ^#^ *p* ≤ 0.01 vs. baseline. *p ≤ 0.05 vs. CPA-control group. NS, not significant.

Baseline hemodynamics were similar in both groups (**Table 2**). No significant difference was observed in groups A and B. Hemodynamic measurements were done to evaluate LV performance using the Millar catheter system in both groups at baseline and after 2 h of reperfusion (**Figure 2**).

**Table 2:** Comparison of baseline hemodynamics in Group A and Group B.

Variables	Group A *n* = 15	Group B *n* = 15	*p* value
HR/min	370	326	Ns*
MAP mmHg	130	129	Ns
LVESP mmHg	95.2	92.2	Ns
LVEDP mmHg	8.2	8.0	Ns
dP/dt max mmHg/s	7,305	7,427	Ns
dP/dt min mmHg/s	7,672	7,511	Ns

*Ns denotes not significant.

The LVESP was improved after 2 h but statistically did not reach the level of significance (*p* = 0.06). The LVEDP measurements showed a reduction after 2 h of reperfusion in the treated versus control group (*p* = 0.03). Ventricular systolic performance dP/dt max after CPB was improved in the FTY720-treated versus control group but did not reach statistical significance (*p* = 0.2). On the other hand, the minimal pressure relaxation rate (dP/dt min) was significantly low in control versus treated group (*p* = 0.02) (**Figure 2**).

### Serum Levels of Inflammatory Mediators

**Figure 3:**
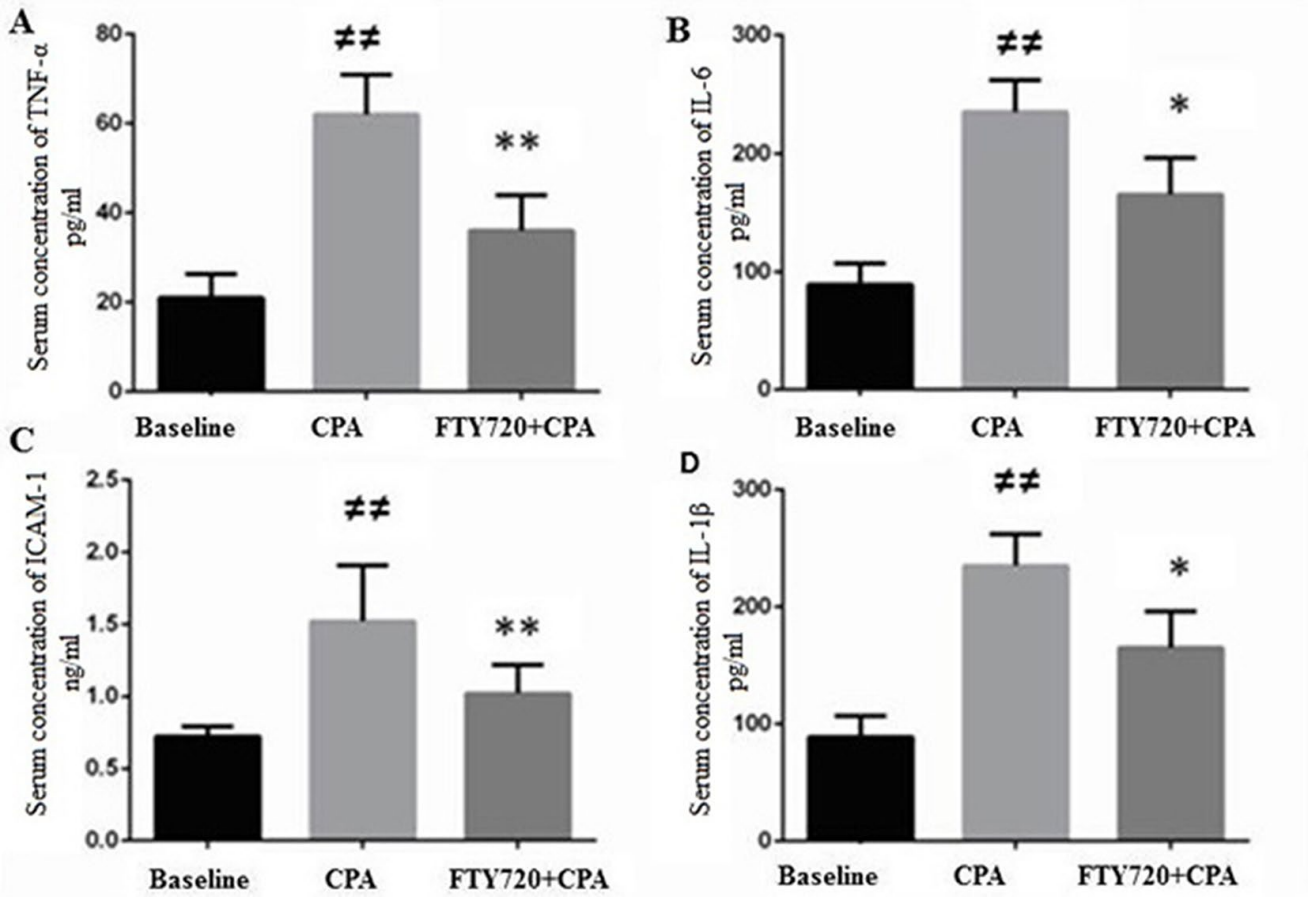
Myocardial production of TNF-α **(A)**, IL-6 **(B)**, ICAM-1 **(C)**, and IL-1β **(D)**. **(A)** CPA model without FTY720 treatment shows a high expression of TNF-α as compared to FTY720 treatment. **(B)** CPA-reperfusion induced significantly high ICAM-1 after 2 h of reperfusion compared with the FTY720-treated and baseline. **(C)** FTY720 treatment remarkably reduced the production of ICAM-1 as compared to control. **(D)** This section of the panel presents the production of 1L-1β, which was higher in the control versusthat in the FTY720-treated group. Each bar height represents the mean ± SD (each group *n* = 15). ^#^ *p* ≤ 0.05 and ^##^ *p* ≤ 0.01 versus baseline. *p ≤ 0.05 and **p ≤ 0.01 versus CPA-control group.

Inflammatory mediators have been described to contribute in the CPA–reperfusion-related I/R injury (Suleiman et al., 2011). In the present study, serum levels of a few important inflammatory mediators were measured. Although ICAM-1 is independently increased in response to I/R, in CPA-related I/R, an increase has been observed. In comparison to baseline, TNF-α, IL-6, IL-1β, and ICAM-1 serum levels were significantly high in the CPA–reperfusion group (*p* ≤ 0.001). On administration of FTY720 (1 mg/kg), TNF-α, IL-6, 1L-1β, and ICAM-1 (vs. control group) were attenuated (*p* ≤ 0.001, *p* = 0.04, *p* = 0.01, and *p* = 0.001), respectively, as shown in (**Figure 3**).

### Nitro-Oxidative Stress

**Figure 4:**
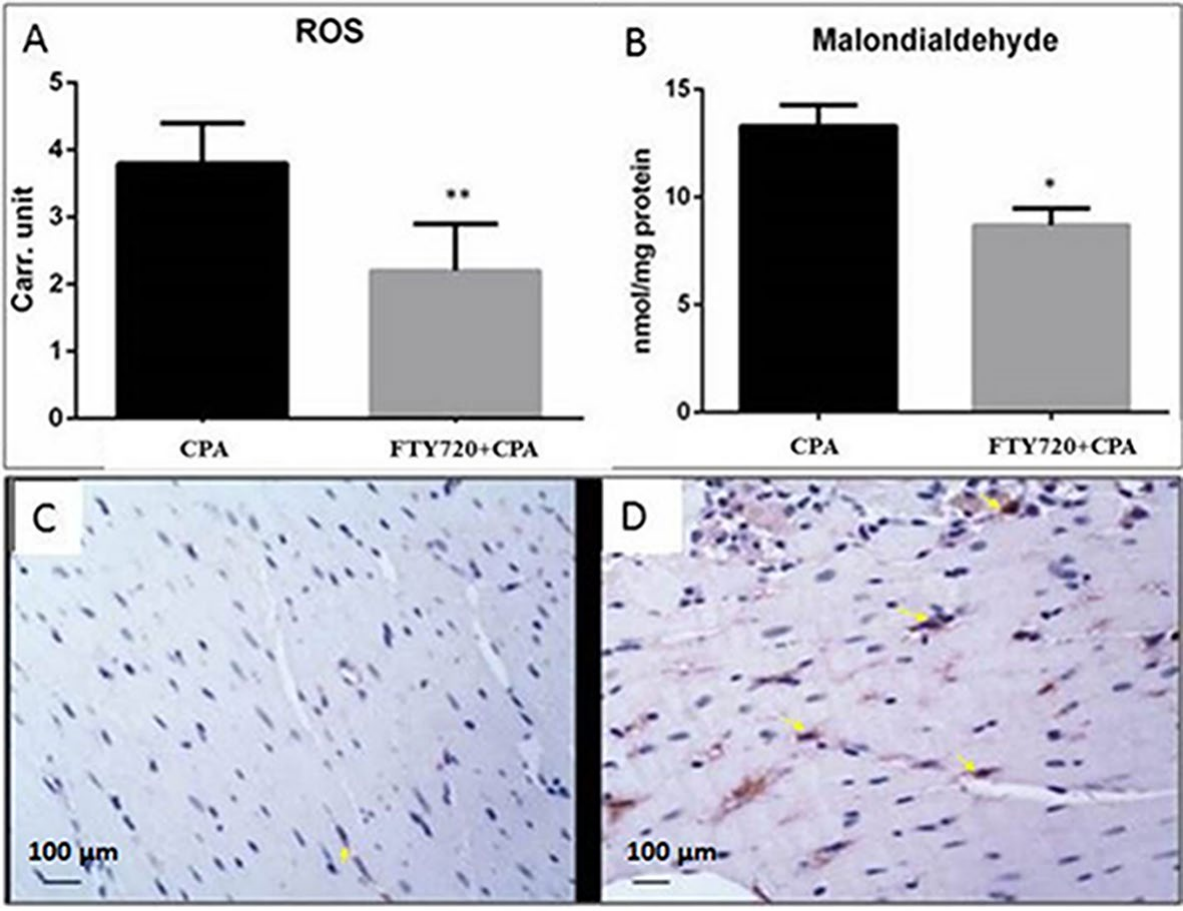
Oxidative stress. Comparison of oxidative and nitrosative stress in FTY720-treated and control group in cardiopulmonary bypass model with CPA **(A** and **B)**. ROS, reactive oxygen species; Carr. unit, Carratelli unit. **p ≤ 0.001, *p ≤ 0.05. Myocardial nitrotyrosine staining **(C)** FTY720-treated group, **(D)** control group (*n* = 15). Rats were subjected to 30 min CPA followed by 2 h reperfusion. Yellow arrows indicate nitrotyrosine expression.

To examine the role of FTY720 in the regulation of free radical production inI/R, we quantified free radicals and aldehydes (lipid peroxidation derivatives) in perfused frozen myocardial samples. In the CPA control group, ROS and MDA levels were higher than those in the CPA-FTY720-treated group. Collectively, our results suggest that FTY720 treatment in CPA decreases oxidative stress (**Figures 4A, B**). Also, treatment with FTY720 resulted in markedly reduced nitrotyrosine protein content, indicative of reduced peroxynitrite generation (**Figures 4C, D**).

### Effect of FTY720 on Erk1/2 and Akt1/2 Signaling Pathways

**Figure 5:**
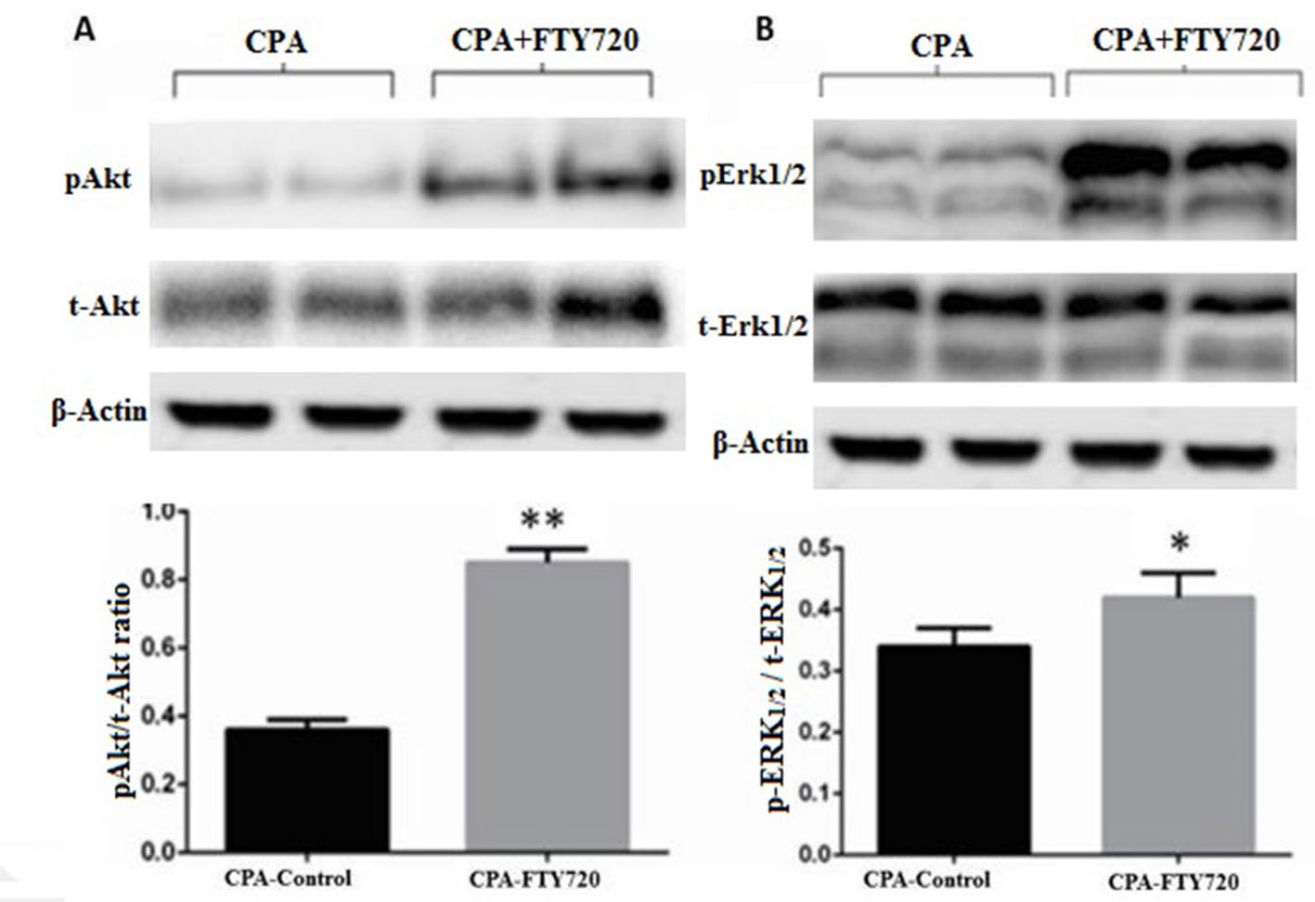
Representative Western blot and relative density ratio of the phosphorylated (p) form of Akt1/2 **(A)** and ERK1/2 **(B)**, samples of left ventricle at the 2-h reperfusion. Relative densities show that FTY720 activates phosphorylation of these proteins. Values are means ± SD; *n* = 15 samples/group. *p ≤ 0.05, **p ≤ 0.001.

Activation of prosurvival signaling pathways (MEK/ERK and PI3K/Akt) was analyzed by measuring the phosphorylation status of ERK1/2 and Akt on Western blots. As shown in **Figures 5A, B** the phosphorylation levels of ERK1/2 and Akt were increased in the treated group compared to those in the controls (*p* ≤ 0.001 and *p* = 0.04, respectively) (*n* = 15 each).

### Bcl-2 and Bax Signaling Pathways

**Figure 6:**
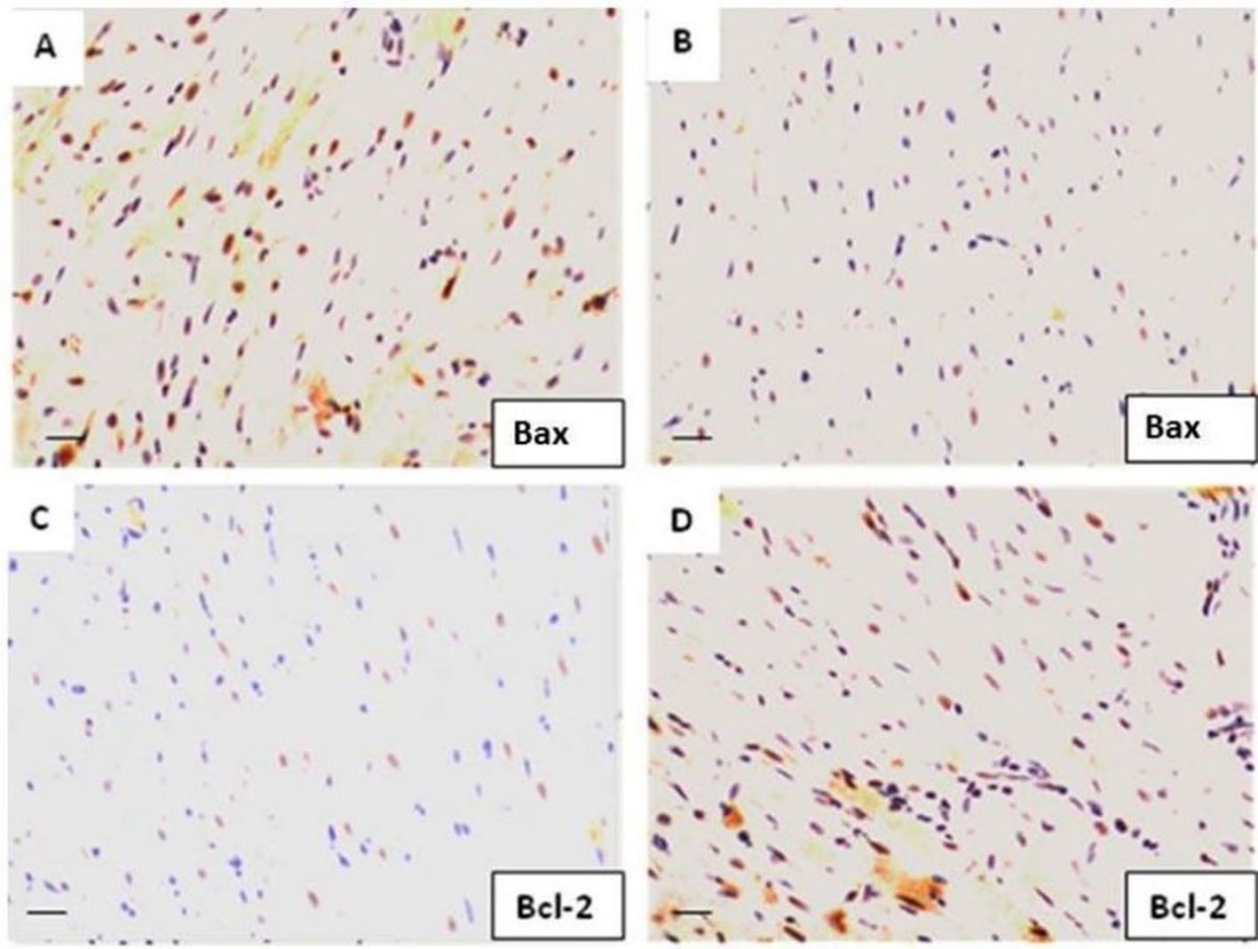
The protein expression levels of Bcl-2 and Bax in myocardial tissue were determined by immunohistochemistry. **(A)** Bax expression in CPA-control group. **(B)** Bax expression in CPA-FTY720-treated group (control vs. treated, *p* ≤ 0.05). **(C)** Bcl-2 expression in the control-CPA group and **(D)** Bcl-2 expression in the FTY720-treated group (control vs. treated, *p* ≤ 0.01).

After 30 min of CPA and 2 h of reperfusion, heart tissue was removed for immunohistological staining to measure the expression of antiapoptotic protein Bcl-2 and its regulator Bax protein. As depicted in **Figures 6A−D**, in the FTY720-treated groups, Bcl-2 expression was significantly increased whereas in the CPA-related I/R group, the expression of Bax was downregulated (*p* = 0.02) but Bcl-2 was upregulated compared to those in Group A. These results indicate that FTY720 during global I/R attenuates apoptosis by upregulation of Bcl-2 and downregulation of Bax proteins.

### Bcl-2 and Bax mRNA Expression Levels

**Figure 7:**
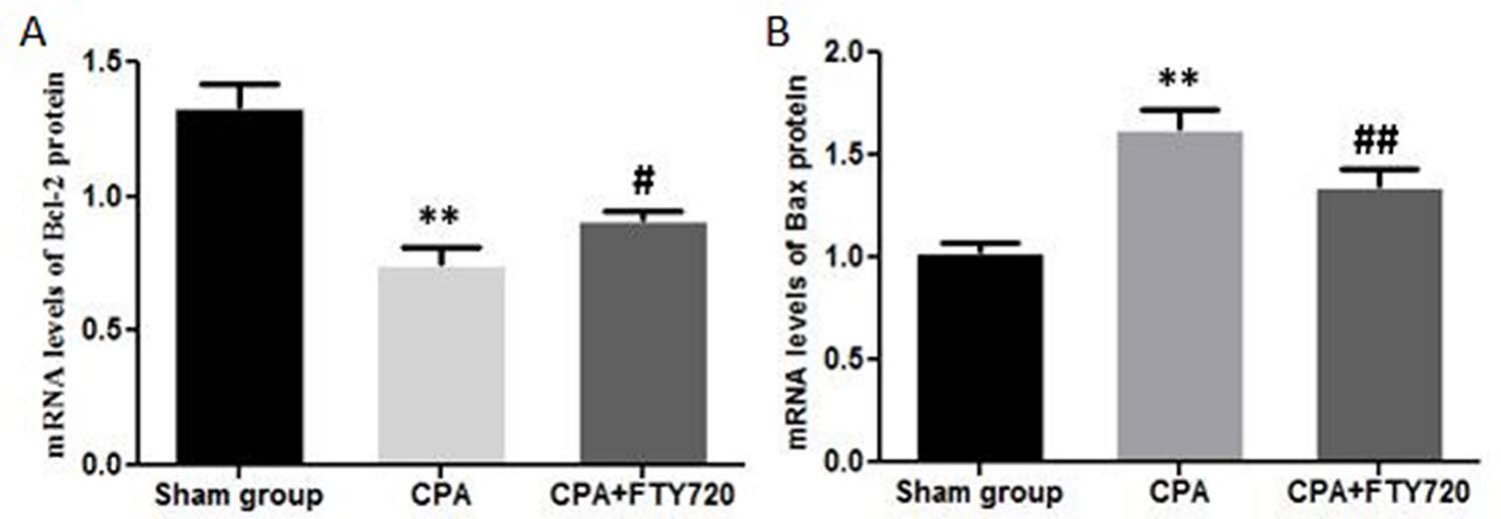
RT-PCR measurement of Bax and Bcl-2 mRNA expression in myocardial tissue showing the FTY720 effect. **(A)** Bcl-2 mRNA expression. **(B)** Bax mRNA expression. **p < 0.01 versus the control group; ^##^ *p* < 0.01 versus the model group and ^#^ *p* < 0.05.

Treatment with FTY720 in the CPA model significantly enhanced the mRNA expression of Bcl-2 and reduced the levels of Bax protein. There was a marked difference in control group as compared to sham group for both proteins. This effect was encountered by fingolimod treatment by decreasing Bax and increasing Bcl-2 levels to limit apoptosis (**Figure 7**).

### High-Energy Phosphates

**Figure 8:**
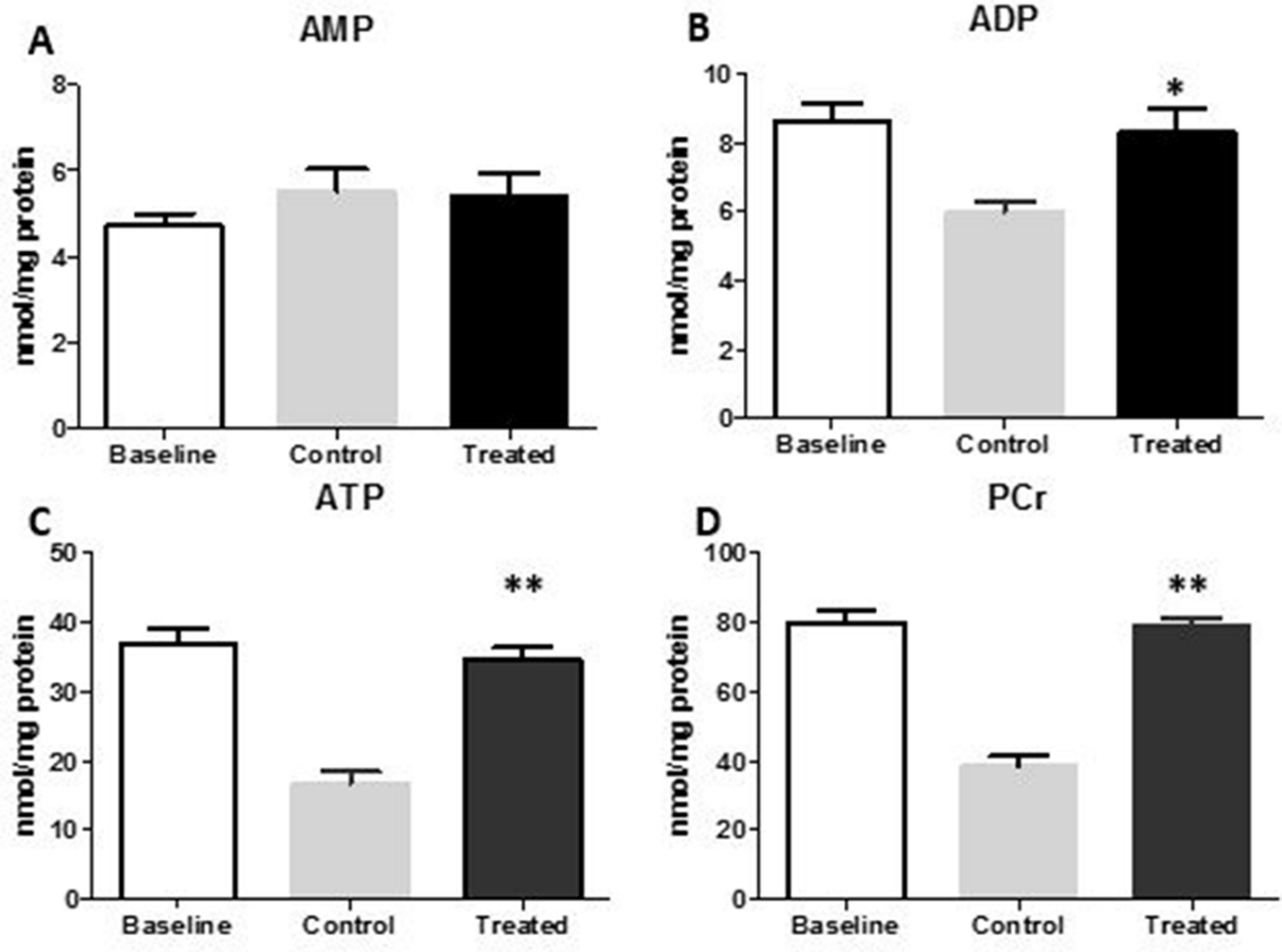
High-energy phosphates in the myocardial tissue of the LV in the FTY720-treated groups compared with those in the control group. **(A**, **B**, **C)** AMP, ADP, and ATP levels after 2 h of reperfusion in CPA. **(D)** Changes in PCr. *p ≤ 0.05, **p ≤ 0.001. AMP, adenosine monophosphate; ADP, adenosine diphosphate; ATP, adenosine triphosphate; PCr, phosphocreatine.

The measurement of high-energy phosphates in the model of CPA revealed the better preservation of FTY720-treated myocardium (**Figures 8A−C**). The AMP levels were similar to control and baseline, but ADP and ATP was significantly raised after 2 h of reperfusion in the treated group as compared to control group (*p* < 0.05, *p* < 0.01, respectively). PCr, the buffering energy source that can be utilized during energy demand in the form of ATP, was significantly higher in the FTY720-administered group compared with that in the control group tissue (*p* < 0.01) (**Figure 8D**).

### FTY720 Attenuates Neutrophil Infiltration

**Figure 9:**
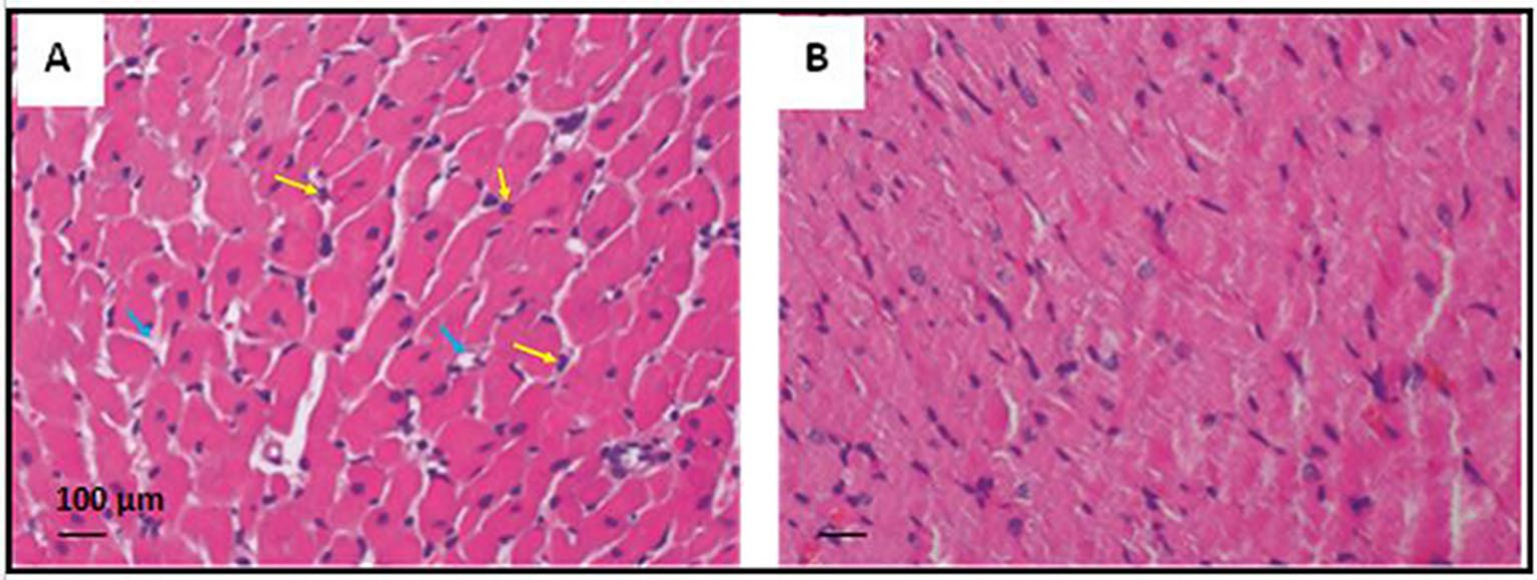
Representative photomicrograph showing the histopathological changes in rats’ myocardium related to CPA-induced ischemia-reperfusion (20X magnification). **(A)** The cardiac section shows interstitial edema and neutrophil infiltration in the control group of CPA rat model. **(B)** Heart tissue section showing reduced edema and neutrophil infiltration in the FTY720-treated group. Yellow arrows indicate neutrophil infiltration, and blue indicates interstitial edema.

In CPA-related I/R, development of interstitial edema, structural disarray, and neutrophil infiltration were observed. However, pre-ischemia FTY720 treatment remarkably reduced morphological changes and neutrophil infiltration, as shown in **Figure 9**.

## Discussion

This study is the first of its kind to investigate the cardioprotective role of FTY720 in a clinically relevant experimental model of cardiopulmonary bypass and CPA, mimicking cardiac surgery settings. The pharmacological cardioprotective strategy to prevent acute global I/R injury has been tested using different approaches. Over the last few years, multiple pharmacological agents, including volatile anesthetic agents. During last decade, different drugs including (Kato and Foex, 2002; Symons and Myles, 2006; Yu and Beattie, 2006), sodium hydrogen exchange inhibitors (Avkiran and Marber, 2002), statins (Lee et al., 1995; Pan et al., 2004; Belhomme et al., 2000), and anti-inflammatory drugs have been investigated as a potential myocardial protective therapies. However, the majority of preclinical strategies showing cardioprotective effects did not show any promising results in clinical settings (Jones et al., 2015).

In this study, administration of FTY720 demonstrated reduced apoptosis by inhibiting inflammation and oxidative stress. Our data show myocardial preservation at both molecular and protein levels. We have also demonstrated the activation of survival signaling pathways, in addition to inhibition of proapoptotic antibodies and activation of antiapoptotic proteins. Akt1/2 and Erk1/2 phosphorylation are important for cell survival pathways, also investigated in this study (Brewster et al., 2012; Bader et al., 2014; Yu et al., 2015).

Previous studies had suggested the cardioprotective role of FTY720 by activating survival pathways in *in vitro* and *ex vivo* models of mouse and rat hearts and *in vivo* animal models (Jin et al., 2002; Lecour et al., 2002). Recently, a study has reported that knockout mice of S1P receptors produced a high level of myocardial damage as compared to wild type (Means et al., 2007). Means et al. (Means et al., 2007) showed that mice lacking sphingosine kinases were found to have a large infarction size compared to control mice. In addition to activation of S1P receptors, the metabolism of S1P showed significance in preconditioning and postconditioning cardioprotective mechanisms (Lecour et al., 2002; Jin et al., 2004; Jin et al., 2007; Gomez et al., 2011).

I/R injury can be caused by various mechanisms and pathways. Activation of apoptotic pathways, complement system activation, increased inflammation, and oxidative stress can cause myocardial injury after I/R (Aletras et al., 2006). FTY720 shows a potential to deal efficiently with most of the myocardial damaging mechanisms and prevent I/R injury.

According to literature, S1P receptor agonists have an important role in immune suppression (Luessi et al., 2015). Various experimental models have been used to test for including a porcine model of I/R (Santos-Gallego et al., 2016) and spontaneous obstructive coronary atherosclerosis murine model (Xu et al., 2014). Using these models, a reduction in infarct size and low mortality have been shown in the FTY720-treated groups. We have measured inflammatory markers in both blood and tissue. In blood, we found a significantly low concentration of neutrophils, lymphocytes, and proinflammatory cytokines.

The ICAM-1, IL-6, IL-1β, and TNF-α as proinflammatory cytokines contribute to the development of inflammatory mechanisms (Bonney et al., 2013; Li et al., 2013; Markowski et al., 2013). A correlation was found between the anti-inflammatory effects of FTY720 on cardioprotection. I/R increased ICAM-1, IL-6, IL-1β, and TNF-α levels in the control group, whereas FTY720 treatment decreased the concentrations of these cytokines. Therefore, the suppression of inflammatory cytokines by FTY720 treatment protects the myocardium from I/R injury caused by these proinflammatory cytokines.

One of the main targets of this drug is to mitigate apoptosis in I/R. Molecular signaling RISK and SAFE pathways activation has been reported during I/R (Lecour et al., 2002; Zhang et al., 2007). The RISK (Akt1/2, Erk1/2, and GSK 3β) and SAFE pathways (JAK and STAT3) are the main sources for the mitigation of apoptosis to prevent the opening of the mPTP (Heusch et al., 2011; Heusch, 2013; Heusch, 2015). Consistent with previous findings, activation of RISK and SAFE signaling pathways was observed after a decreased level of apoptosis in the treated group as compared to that in the control. The decreased activation of proapoptotic proteins Bax and enhanced immunoreaction for antiapoptotic protein Bcl-2 was observed after 30 min of CPA and 2 h of reperfusion. Reperfusion after transient ischemia in the myocardial tissue leads to cardiac apoptosis and reduced myocardial function (Khan et al., 2006; Oh et al., 2013). That shows the protective effect of role of FTY720 by reducing inflammatory mediators and oxidative stress. Considering the outcome of our study, we believe that the investigation of S1P receptor activation in patients undergoing CPA during cardiac surgery will be helpful to assess the potential clinical efficacy of this cardioprotective role.

## Limitations

According to literature, coronary artery disease will be the most common cardiovascular disease having highest mortality rate by 2020 in the entire world by 2020 (Yellon and Baxter, 2000). Although with advances in percutaneous techniques, majority of the patients undergo percutaneous coronary intervention (PCI) but coronary artery bypass grafting ( CABG) remains the mainstay of treatment in complex coronary artery disease and/or valvular disease. Coronary artery disease patients undergoing cardiac surgery mostly have associated comorbidities, including diabetes, hypertension, and hyperlipidemia, whereas in our experimental model, it was not all truly reflected.

In our study, reperfusion time was 2 h, and we could not study the effect of FTY720 in late phase. Prolonged reperfusion may give more strong evidence of reduction in I/R injury. The effect of FTY720 in structural remodeling can be studied only if we could increase our reperfusion time but because of the complexity of surgery, it is hard to practice.

Furthermore, this study did not assessed the capability of FTY720 to enhance myocardial salvage with multiple doses of FTY720 or its chronic use because we administered a single dose before initiating ischemia in our model.

## Conclusion

In conclusion, FTY720 preserves high-energy phosphates and cardiac mechanical function, as well as attenuation of myocardial apoptosis, inflammation, and oxidative stress. Furthermore, a cardioprotective mechanism is associated with phosphorylation of Akt1/2 and Erk1/2 prosurvival cell signaling pathways to prevent myocardial damage. Moreover, the question whether a lower dose will have protective effects on the myocardium in humans needs to be answered in further studies. Future studies of prolonged reperfusion with pre-ischemia and post-ischemia and treatment with FTY720 may give better results and enable the analysis of the effect on myocardial remodeling.

## Data Availability

The datasets generated for this study are available on request to the corresponding author.

## Ethics Statement

This study was conducted in the Division of Cardiac Surgery and Translational Surgery Lab, University of Verona Medical School, Verona, Italy. The study was directed in compliance with best research practice and according to the Declaration of Helsinki principles. The protocol was approved by the local Joint Ethical Committee for University of Verona and Hospitals (Verona and Rovigo), Italy. (BBCCH1337).

## Author Contributions

NA: Design, experimental work, bench work and manuscript writing; SS: Bench work; AM: Data analysis; DL, MT: Experimental work; NM: Statistical analysis; AG, MI: Manuscript writing and critical review; RR: Data analysis; SM, WH: Data analysis; GF: Supervised and reviewed experimental protocol, AR: Designed protocol, supervised, conducted experimental work.

## Funding

This study was financially supported by Deanship of Scientific Research, King Saud University, Riyadh, Saudi Arabia, which supported the work through the research group (RGP-VPP 181), Cardiac Surgery Division, University of Verona, Verona, Italy, and the Department of Biological and Biomedical Sciences, Aga Khan University, Karachi, Pakistan.

## Conflict of Interest Statement

The authors declare that the research was conducted in the absence of any commercial or financial relationships that could be construed as a potential conflict of interest.
